# Thermal-Performance Instability in Piezoresistive Sensors: Inducement and Improvement

**DOI:** 10.3390/s16121984

**Published:** 2016-11-24

**Authors:** Yan Liu, Hai Wang, Wei Zhao, Hongbo Qin, Xuan Fang

**Affiliations:** 1 School of Electro-Mechanical Engineering, Xidian University, Xi’an 710071, China; liuy@xidian.edu.cn (Y.L.); weizhao@xidian.edu.cn (W.Z.); qhb0920qhb@xidian.edu.cn (H.Q.); fangxuan21@163.com (X.F.); 2 State Key Laboratory for Manufacturing Systems Engineering, Xi’an Jiaotong University, Xi’an 710054, China

**Keywords:** piezoresistive sensor, thermal-performance instability, inducement, improvement

## Abstract

The field of piezoresistive sensors has been undergoing a significant revolution in terms of design methodology, material technology and micromachining process. However, the temperature dependence of sensor characteristics remains a hurdle to cross. This review focuses on the issues in thermal-performance instability of piezoresistive sensors. Based on the operation fundamental, inducements to the instability are investigated in detail and correspondingly available ameliorative methods are presented. Pros and cons of each improvement approach are also summarized. Though several schemes have been proposed and put into reality with favorable achievements, the schemes featuring simple implementation and excellent compatibility with existing techniques are still emergently demanded to construct a piezoresistive sensor with excellent comprehensive performance.

## 1. Introduction

Piezoresistive sensors were one of the first developed microelectromechanical system (MEMS) devices and still display a significant growth prompted by the advancements in surface/bulk micromachining techniques and material technology [1,2,3]. Until now, silicon piezoresistances have been utilized in the detection of various targets, including acceleration [4,5,6], pressure [7,8], micro force/torque [9,10], strain/stress [11,12], flow [13,14], biochemical interaction [15,16], fluid density and viscosity [17,18], surface topography [19], sonar vectors [20], etc.

**Figure 1:**
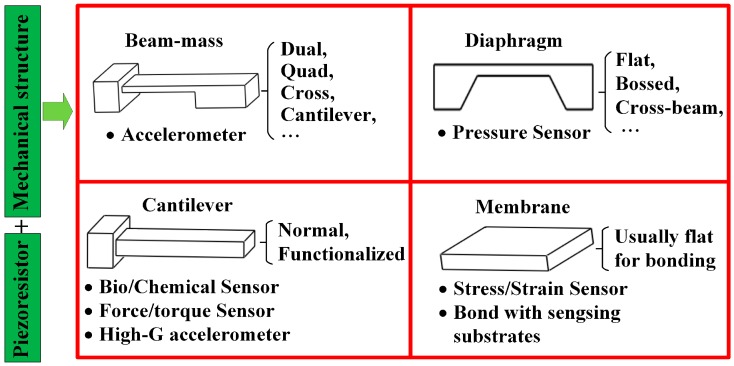
Classic structures for piezoresistive sensors.

To fulfill the demands from different applications, various classic structures for piezoresistive sensors, as shown in Figure 1, are proposed and proved to be high effective by practice. As the main geometrical configuration for accelerometers, the beam-mass structure can work smoothly by sensing the inertia force caused by the applied acceleration. Single cantilever-mass structures, which appeared in the first monolithic microaccelerometers, feature good sensitivity but poor cross-axis immunity and dynamical response [21]; the dual cantilever-mass structure partly solves the latter problem by promoting the beam-perpendicular-direction stiffness; subsequently, the double-supported-beam, quad-beam and cross-beam structures greatly enhance the primary and lateral stiffness, but diminish the sensitivity at the same time [22]; then, many improved sensing structures (e.g., multi-beam-mass [23], slotted-beam-mass [24], planner-beam [25], gapped-cantilever-beam [26] etc.) are constructed to obtain better comprehensive characteristics considering the sensitivity, resonant frequency and cross-axis sensitivity. Flat and bossed diaphragms are the most used schemes for pressure sensors. Flat diaphragms with different geometry (e.g., square, circle and rectangle) and thickness (from several microns to hundreds of microns) can measure pressures from Pa to MPa with favorable sensitivity and dynamic response [27,28]; the bossed diaphragms provide the sensor with better linearity and overload resistance when used in ultra-high sensitive measurement of vacuum levele [29,30]; meanwhile, cross-beams, peninsula-islands and annular grooves are also incorporated into the flat or bossed diaphragms to further optimize the sensor features [31]. Cantilevers are often utilized as a probe in micro-force detection, atomic force microscope measurement, chemical or bio-sensing, and some accelerometers also adopt cantilevers to acquire high-G acceleration signals [15,32]. Stress/strain sensing membrane can be bonded onto various substrates to construct specific sensing devices, such as the GPa-level pressure sensor [33], force sensor for cutting force/torque in turning and milling [34,35].

Benefiting from the advantages of a mature process, diversity in structural composition, simple signal transduction and conditioning, and favorable performance, piezoresistive sensors can be found in nearly every domain, from automobiles, interstellar probes, and consumer electronics to human health monitoring and infant nursing [1,36,37]. However, these widely applied devices often suffer from performance deterioration induced by externally loaded thermal or temperature effects, because of the intrinsic temperature dependence of silicon piezoresistance and their stress-sensitive mechanism. Firstly, the resistance of piezoresistors and piezoresistive coefficient will vary with the temperature, which will influence the zero-offset and sensitivity of the device [38]. Then, as a stress-based transduction device, the residual stresses from the chip fabrication process [39,40], wafer bonding [41], adhesion and compound molding [42,43] can lead to obvious variations in sensor parameters. Meantime, the difference of coefficient of thermal expansion (CTE) between the materials will induce a significant accompanying stress in the device, and the effect varies under different ambient temperatures for different sensors [44,45,46].

In the past few years, several good reviews on piezoresistive pressure sensors, force sensors and accelerometers have been published. Doll et al. [47] have published a book about piezoresistor design and applications based on a review published in 2009 [1]. The main purpose of this book is to address the need for a comprehensive guide to piezoresistor design and the analysis of thermal effects. However, the work only concentrates on the intrinsic temperature dependence of piezoresistors, without mentioning the influence of residual stress. Liu et al. [22] focused on geometrical improvements for enhancement of accelerometer sensitivity, resonant frequency and cross-axis sensitivity, but the thermal stability was not discussed. Kumar et al. [48] presented the design principles and considerations for favorable pressure sensors. In that review, the dependence of piezoresistive coefficient on temperature, zero-offset and its temperature drift were described. Some conventional compensation methods, e.g., two Wheatstone bridge counteraction, were provided. Obviously, there is a distinct lack of a systematic and thorough overview of the thermal-performance stability of piezoresistive sensors. This review aims to survey the inducements of thermal-performance instability, available enhancement strategies and their emerging applications. The paper is organized as follows: first, the fundamentals of piezoresistive sensors are described in Section 2. Herein, we summarize the operation mechanism of piezoresistive devices, and silicon piezoresistance is also mentioned. Second, inducements of thermal-performance instability are presented in Section 3. The piezoresistive coefficient, chip fabrication processes and device packaging are all investigated to conclude the mode of influence of different parameters on sensor performance. Section 4 summarizes the available methods for the improvement of thermal stability. Finally, conclusions are proposed in Section 5.

## 2. Fundamentals of Piezoresistive Sensors

**Figure 2:**
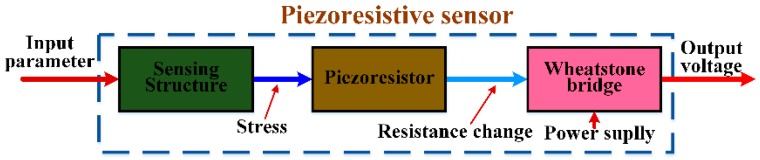
Basic principle for piezoresistive sensors.

A piezoresistive sensor is a category of sensor that acquires target parameters and transforms them into piezoresistance changes. The basic principle is illustrated in Figure 2. Firstly, the input parameter acts as a kind of mechanical load onto the sensing structure and is transduced into a stress-induced resistance change of the embedded piezoresistor. Then, the resistance change is converted to an output voltage of measurement circuit, typically a Wheatstone bridge. The output voltage may be conditioned by amplifiers and acquired by a multimeter. Depending on the different targets being detected, the sensing structure varies, as mentioned in the section above.

The fundamental principle here is the piezoresistive effect, which refers to the phenomenon that the electrical resistance changes in response to mechanical stress. The piezoresistive effect in semiconductors, especially in silicon, is widely used in various sensors since it was first reported by Smith in 1954. Generally, the relative change in resistance (Δ*R*/*R*) of a material can be written as:

(1) $$\frac{\Delta R}{R}=\frac{\Delta \rho }{\rho }+(1+2\upsilon )\varepsilon =\pi \sigma +(1+2\upsilon )\varepsilon =(\pi E+1+2\upsilon )\varepsilon =G\varepsilon$$

where *ρ* is the resistivity, *ν* is the Poisson’s ratio, π is the piezoresistive coefficient and *ε* is the strain, *G* is the gauge factor. Herein, the resistance change can be regarded as two terms: the one due to changes in resistivity and the other due to changes in geometry. For silicon and other semiconductors, the first term, Δ*ρ*/*ρ*, can be 50–100 times larger than the second one because of their piezoresistive effect. Moreover, the relationship between the applied stress *σ_λ_* and resistivity in a fixed current orientation (*i*) can be expressed as:

(2) $$\frac{\Delta \rho_{i}}{\rho }=\sum_{\lambda =1}^{6}\pi_{i\lambda }\sigma_{\lambda }$$

where *π_iλ_* is the piezoresistive coefficient in the corresponding orientation. Though the piezoresistive coefficient varies among the crystallographic orientation, the effect on resistors is more of concern when designing a piezoresistive sensor. Based on the axes transformation method by Bao and neglecting the geometrical term in Equation (1), the relative change in resistance can be written as a combination of the longitudinal and transverse components as:

(3) $$\frac{\Delta R}{R}=\pi_{l}\sigma_{l}+\pi_{t}\sigma_{t}$$

where *π_l_*, *π_t_* are the longitudinal, transverse piezoresistive coefficients, and *σ_l_*, *σ_t_* are the longitudinal and transverse stresses. For p-silicon piezoresistors aligned along the <110> orientation on (100) wafers, Equation (3) can be further nailed down as:

(4) $$\frac{\Delta R}{R}=\frac{1}{2}\pi_{44}(\sigma_{l}-\sigma_{t})$$

where the components of piezoresistive coefficient tensor in silicon *π*_11_ and *π*_12_ are neglected, and *π*_44_ = 138.1 × 10^−11^ Pa^−1^. With the abovementioned expressions, the relative change in resistance of a piezoresistor under a certain stress filed can be calculated easily.

**Figure 3:**
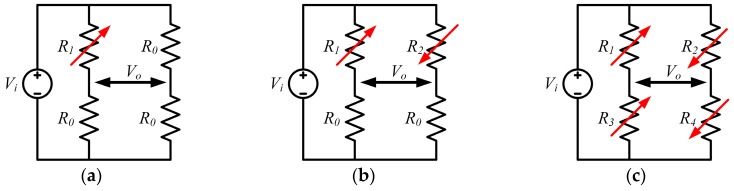
Different configurations of Wheatstone bridge in piezoresistive sensors. (**a**) Quarter bridge; (**b**) Half bridge; (**c**) Full bridge.

**Figure 4:**
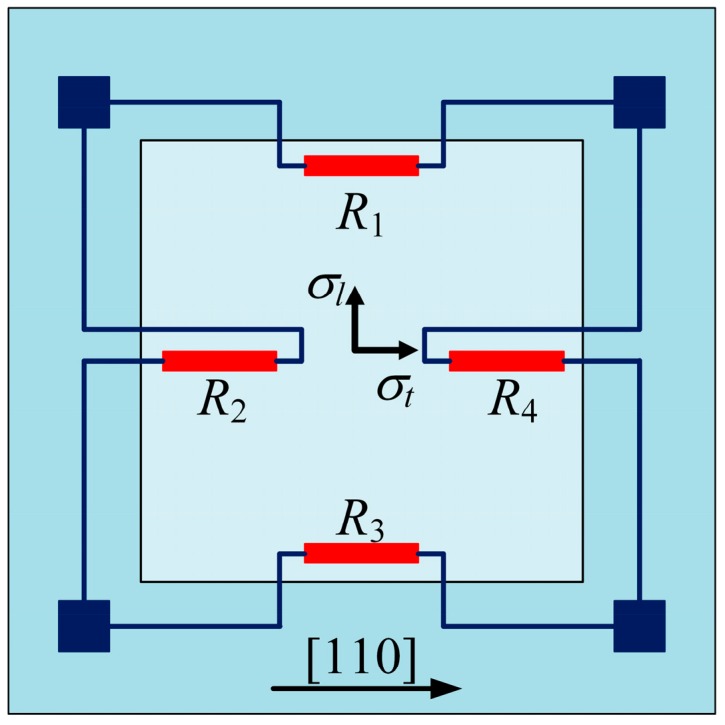
Piezoresistors distributed on the top of diaphragm in a pressure sensor.

The most common measurement circuit for relative resistance change is the Wheatstone bridge, where one or more mechanically loaded piezoresistors are arranged to form a quarter, half or full bridge configuration, as shown in Figure 3. Among them, the full bridge configuration has the best sensitivity, as the value of a quarter bridge is only 25% of that of the full bridge. The Wheatstone bridge can be excited with either a voltage or current source. Herein, we take a full bridge excited with a voltage source *V_s_* as investigation object. The piezoresistors in the bridge are distributed on the top of a diaphragm as Figure 4 shows. Every resistor experiences both longitudinal and transverse stress. Because of the symmetry in the diaphragm structure and the piezoresistors’ location, the longitudinal *σ_l_*, and transverse *σ_t_* stress experienced by *R*_1_ and *R*_3_ are also the transverse and longitudinal stress experienced by *R*_2_ and *R*_4_. At the initial state, *R*_1_ = *R*_2_ = *R*_3_ = *R*_4_ = *R*_0_, and resistance change Δ*R_i_* is very small when compared with *R*_0_. The output voltage *V_o_* of the full bridge sensor can be given by:

(5) $$\begin{array}{cc} V_{o} & =\frac{1}{4}(\frac{\Delta R_{1}}{R_{1}}-\frac{\Delta R_{2}}{R_{2}}+\frac{\Delta R_{3}}{R_{3}}-\frac{\Delta R_{4}}{R_{4}})V_{s}=\frac{1}{2}(\frac{\Delta R_{1}}{R_{1}}-\frac{\Delta R_{2}}{R_{2}})V_{s}=\frac{1}{2}\left[\frac{1}{2}\pi_{44}(\sigma_{l}-\sigma_{t})-\frac{1}{2}\pi_{44}(\sigma_{t}-\sigma_{l})\right]V_{s}\\ & =\frac{1}{2}\pi_{44}(\sigma_{l}-\sigma_{t})V_{s} \end{array}$$

Obviously, it is the differential stress (*σ_l_*−*σ_t_*) sensed by piezoresistors that determines the sensitivity of piezoresistive sensors, and any stress fluctuation will decrease the device accuracy and lead to performance instability, especially for the mutable applications with variable temperature. In the following section, the instability induced by thermal/temperature will be discussed in detail.

## 3. Inducements to Thermal-Performance Instability

**Figure 5:**
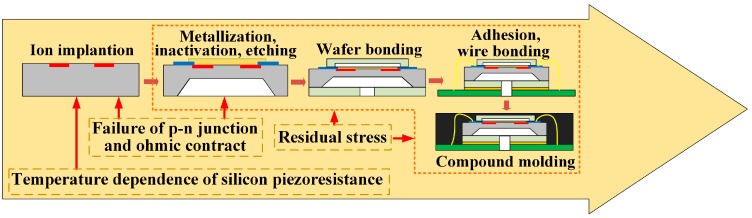
Possible instability of piezoresistive sensor in the main manufacturing processes.

Thermal-performance instability is the most ubiquitous obstruction in the application of piezoresistive sensors. The related thermal characteristics have drawn a great attention since the utilization of silicon piezoresistance in sensing devices. There are mainly three sources of performance perturbation in the fabrication and packaging of piezoresistive sensor, including temperature dependence of piezoresistive coefficient and resistance, residual stress in the passivation/metallization layers, chip adhesion and compound molding. Meanwhile, the p-n junction and ohmic contract (especially for aluminum) may fail at high temperature conditions. The instability appears from the beginning of sensor chip fabrication as illustrated in Figure 5. Each inducement features its own affection mode and intensity. Thus, it is very important for the elimination of interference effect that to get an insight into the sources of thermal-performance instability. In the following parts, the principle and affection mode of these inducements will be reviewed and summarized.

### 3.1. Influence of Silicon Piezoresistance

As we all know, the large piezoresistive effect of silicon derives from the stress-related changes in the band diagram and effective mass of holes and electrons, which leads to a change in the resistivity. However, the number of carriers is also sensitive to the temperature variation. Inevitably, the resistivity and piezoresistive coefficient will vary under different temperatures, which in turn causes the change in sensor zero-offset and measurement sensitivity.

Considering the operation temperature *T* and stress *σ* loaded by measurement targets, the resistance of each piezoresistor in the Wheatstone bridge can be expressed as (assuming *T_0_* = 0 °C):

(6) $$R_{i}\left(T,\sigma \right)=R_{i,0}+R_{i,0}\left(\alpha_{i}T+\beta_{i}T^{2}\right)+R_{i,0}\pi_{44}\left(1+\delta_{i}T\right)\Delta \sigma_{i}\;\left(i=1,2,3,4\right)$$

where *R_i,0_* is the initial resistance of *i*th piezoresistor at reference temperature *T_0_*, α*_i_*, β*_i_* are the temperature coefficients of resistivity (TCR), δ*_i_T* refers to the temperature-induced variation of piezoresistive coefficient, which mainly determines the temperature coefficient of sensitivity (TCS), Δσ*_i_* is the difference between longitudinal and transverse stress. Thus the zero-offset voltage of the bridge in Figure 4 at *T* can be calculated as [49]:

(7) $$\frac{V_{0,o}\left(T\right)}{V_{s}}=\frac{R_{1,0}R_{2,0}}{{\left(R_{1,0}+R_{2,0}\right)}^{2}}\times \left[\left(\alpha_{1}-\alpha_{2}\right)T+\left(\beta_{1}-\beta_{2}\right)T^{2}\right]-\frac{R_{4,0}R_{3,0}}{{\left(R_{4,0}+R_{3,0}\right)}^{2}}\times \left[\left(\alpha_{4}-\alpha_{3}\right)T+\left(\beta_{4}-\beta_{3}\right)T^{2}\right]$$

Let:

(8) $$A=\frac{R_{1,0}R_{2,0}}{{\left(R_{1,0}+R_{2,0}\right)}^{2}}\;B=\frac{R_{4,0}R_{3,0}}{{\left(R_{4,0}+R_{3,0}\right)}^{2}}$$

Compensating the mismatch of *R_i_*_,0_ is realizable in practical applications. Thus, *A* and *B* would be adjustable and not greatly affect the magnitude of the thermal variations of zero-offset. The zero-offset mainly suffers from the mismatch of TCR in different piezoresistors. If the quadratic term of *T* equals zero, the zero-offset variation in a limited range of temperature will be linear with a positive or negative slop, depending on the sign of calculated results of *α_i_*; if the quadratic term of *T* appears, Equation (7) will turn into a parabola, reaching the maximum offset voltage at the temperature:

(9) $$T_{0m}=\frac{1}{2}\frac{A\left(\alpha_{1}-\alpha_{2}\right)-B\left(\alpha_{4}-\alpha_{3}\right)}{A\left(\beta_{1}-\beta_{2}\right)-B\left(\beta_{4}-\beta_{3}\right)}$$

To simplify the expression of TCS, *R_i_*_,0_ is set equal to each other, and *A* = *B* = 0.25. The output voltage of full bridge with Δσ*_i_* at *T* can be written as:

(10) $$\frac{V_{\sigma ,o}\left(T\right)}{V_{s}}=\frac{1}{4}\sum_{i=1}^{4}{\left(-1\right)}^{i+1}(\alpha_{i}T+\beta_{i}T^{2})+\frac{1}{4}\pi_{44}\sum_{i=1}^{4}\left(1+\delta_{i}T\right)\Delta \sigma_{i}$$

**Figure 6:**
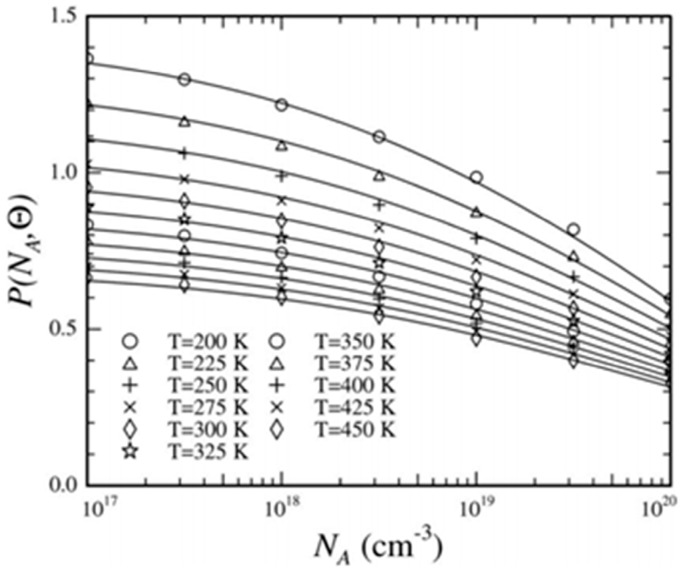
Fitted piezoresistive factor for *π*_44_ as a function of doping concentration and temperature; Reprinted from [51], with permission of AIP Publishing.

The first item is the voltage induced by TCR, and the second one is the effect of temperature dependence of piezoresistive coefficient, which greatly influences the TCS. However, the relationship between the piezoresistive coefficient and temperature is very complex. The dependence of piezoresistive coefficient on temperature and doping concentration is firstly reported by Kanda with the help of theoretical work and detailed experiments [38]. In the model of Kanda, the coefficient can be calculated by multiplying a piezoresistive factor, *P(N_A_,T)*, with the piezoresistive coefficient at the temperature of 300 K. Then, a more particularly useful fitting function of piezoresistive factor for *π*_44_ is proposed by Richter as [50,51]:

(11) $$P(N_{A},\Theta )=\Theta^{-\vartheta }{\left[1+{\left(\frac{N_{A}}{N_{b}}\right)}^{\tau }\Theta^{-\upsilon }+{\left(\frac{N_{A}}{N_{c}}\right)}^{\lambda }\Theta^{-\eta }\right]}^{-1}$$

where *N_A_* is the doping concentration, *Θ* = *T*/*T*_0_ and *T*_0_ = 300 K. Other symbols in Equation (11) are the fitting items which can be found in [51], and the fitting results are shown in Figure 6 together with normalized piezocoefficient values calculated using the 6 × 6 **k·p** Hamiltonian model. *P*(*N_A_*, *Θ*) is larger at lower doping concentration, giving rise to a higher measurement sensitivity. Meanwhile, higher temperature dependence of *P*(*N_A_*, *Θ*) also appears, causing large temperature drift in sensitivity. At higher *N_A_*, there is a drop in the sensitivity but the *P*(*N_A_*, *Θ*) curves of different temperature tend to converge, leading to a less TCS.

Temperature dependence of resistivity and piezoresistive coefficient leads to an obvious shift both in the sensor zero-offset and measurement sensitivity, and the imperfection is an inherent feature in the piezoresistive devices. To construct a robust sensor, the abovementioned effect should be well understood and proper doping parameters should be chosen. Also, necessary compensation measures may help in decreasing temperature drift, which will be discussed in the next section.

### 3.2. Influence from Membrane

As abovementioned, a piezoresistive sensor is a kind of stress-sensing device and the sensor performance will be deeply influenced by the extra stresses along with target-induced ones. Among them, residual stress, often arising in the processes of chip fabrication and packaging, is one of the influential stresses for its thermal hysteresis feature. The membrane often acts as the prerequisite layer (e.g., passivation layer, metallization layer and functional layer) in sensor chip fabrication, and residual stress may arise in its formation.

Passivation is an inevitable process for nearly every MEMS device, and the passivation layer can prevent the existing elements from being damaged in the subsequent processes [52]. Passivation layers, usually made of SiO_2_ and Si_3_N_4_, are often realized by thermal oxidation, plasma enhanced chemical vapor deposition (PECVD) or low-pressure chemical vapor deposition (LPCVD) [53]. However, the passivation layer frequently suffers from process-induced stress, whose state may vary with the layer material and fabrication process. The oxide film undergoes a compressive stress, and its amplitude will increase with the membrane thickness; meanwhile, the nitride film often features tensile stress. When the oxide film covers only one side of the silicon water, excessive bow up to several μms could happen, which will influence the precision of fabrication and sensor performance. Though the stress in oxide film can be compensated by LPCVD nitride film, completely elimination of wafer bow still cannot be achieved. The uncompensated stress may have nearly no effect on a general structure with large dimensions, but still cause an obvious decline in the performance robustness of many devices with thin diaphragms or tiny suspended beams [4,54]. For a diaphragm-based pressure sensor, the residual stress-induced curvature is related to the residual stress by Stoney equation as [55]:

(12) $$\kappa =\frac{6(1-\upsilon_{d})\sigma_{r}}{E_{d}h_{d}^{2}}$$

where *υ_d_*, *E_d_* and *h_d_* are the Poisson’s ratio, elastic modulus and thickness of the diaphragm, respectively; *σ_r_* is the residual stress. Wang and Li investigated the thermal instability of a micro-pressure sensor caused by insulating dioxide and/or nitride film on the top of a silicon diaphragm, whose thickness was much smaller than its width and length [54]. The scanned results showed an approximation between the insulating-layer induced parasitic diaphragm deflection and ANSYS simulated diaphragm deflections under applied pressure, and the approximation disappeared when the insulating layer was removed by thermal phosphate or wet HF treatment. The parasitic deflection would lead to supposititious variation of piezoresistance and change the zero-offset of sensor. The temperature coefficient of zero-offset (TCO) for sensor with passivation layer was 0.023%/°C FS, which was 11 times larger than the value if proper compensation was conducted to get rid of the influence from passivation layer, further confirming the residual stress effect in passivation layer [39].

The metallization layer connects the piezoresistors into Wheatstone bridge and provides an electrical interface between the sensor chip and external components. In spite of their small area, inappropriate arrangement of metal wires and pads can also lead to thermal hysteresis in sensor performance because of the hysteresis stress remained in the metal layer after the heating and cooling process in metal sputtering. Chiou and Chen conducted a detailed study on the thermal hysteresis of a pressure sensor [56]. The results from finite element analysis (FEA) and experiments showed that aluminum (Al) layer on the diaphragm was the potential root cause to the thermal hysteresis problem. Meantime, different layout of Al wires and pads leaded to different hysteresis states. The disparity between them mainly laid on the size and location pads. The improved proposal obtained smaller pads and a more dispersed location arrangement away from the sensor diaphragm. The same results can also be found in the paper by Chiang et al. [57], in which they claimed that the thermal hysteresis of pressure sensor could be reduced by an improved Al layout with trace lines that shorter, more uniform and symmetrical, far away from silicon diaphragm and piezoresistors.

Functional layers are mainly applied in bio/chemical sensors to capture a certain target [58,59]. Caused by the stress in functional layer, the cantilever-based sensor often behaves an excessive deflection and larger zero-offset. However, the bio/chemical sensors usually work in a + temperature, and the stress in functional layer also maintains a stable state, realizing an acceptable stability in the sensor performance [15,60].

### 3.3. Influence from Anodic Bonding

Wafer bonding is a preparatory process for subsequent chip packaging [61,62]. Anodic bonding between glass and Si wafers is widely utilized for wafer level packaging. Anodic bonding is a solid state, field-assisted, irreversible bonding technique. Bonding between silicon and Corning Pyrex 7740 (or Schott Tempax 8330) glass by applying voltage, temperature and/or pressure is the most established scheme [28]. The common parameters for the process include 400–1000 V of voltage and 350–450 °C of temperature. At these higher temperatures, stress may leading to wafer bowing, which is a striking drawback in sensor chip fabrication. Though Pyrex 7740 is specially doped to achieve conductance and a closer CTE to Si at the bonding temperature, the tiny deviation of CTE still cause a stress that cannot be neglected [63]. Meanwhile, the CTE deviation changes with the temperature, bring different stress states to the chip. The CTE of silicon is larger than that of Pyrex 7740 at the temperatures above 315 °C, resulting in a residual tension in the silicon when the bonded wafer pair is cooling down to room temperature. At 400 °C, the difference in CTE between the bonded wafers can be up to about 7%, which creates a wafer deflection with the curvature in the order of tens to hundreds of microns over a 100 mm wafer [64]. Corresponding to this, for silicon and Pyrex wafers with the same thickness, the residual stress in silicon wafer will be tensile if the bonding is carried out above 315 °C, and compressive if the bonding temperature is below 315 °C. Moreover, the nonuniform local contact between the wafers during bonding will cause a varied temperature distribution across the wafer and lead to significant local residual stress. Besides the difference of CTE, the Poisson’s ratio may also be crucial in residual stress. The residual stress in the bonding interface will greatly affect the accuracy and zero-offset of piezoresistive sensors. According the FEA results in [65], the anodic bonding induced stress can result in a zero-offset voltage about 1.9 mV at bonding temperature of 450 °C, and higher temperature will synchronously increase this voltage.

### 3.4. Influence from Chip Adhering and Compound Molding

**Figure 7:**
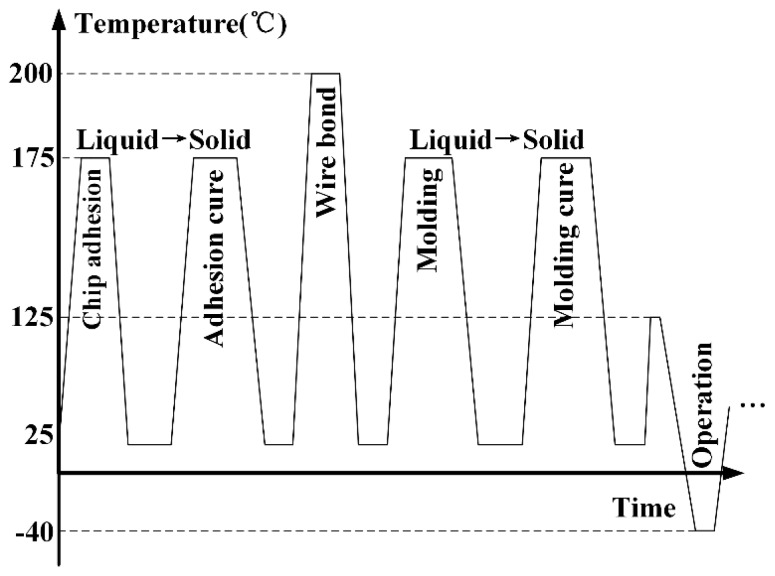
Typical temperature variation during sensor chip packaging, redrawn from [67].

Chip adherence and compound molding involve temperature variation and phase changes of different materials as shown in Figure 7. Residual stress will inevitably appear in the manufactured devices [66]. After cap bonding and singulating from wafer, the sensor chips are then adhered onto the chip board using epoxy adhesive. The adhesion temperature is about 175 °C, and the epoxy turns into solid state to fix the chip. Then, standard wire bonding is conducted to provide electrical connections to the sensor after necessary adhesion cure (at 175 °C), and the bonding temperature is about 200 °C. Compound molding is another important process that prevent the sensor chip from damage. The molding and cure are both operated at 175 °C, and liquid-solid conversion of the used multi-aromatic epoxy resin material also appears. The singulated devices are then brought to various applications and work in the temperature range of −40 °C to 125 °C.

At the molding temperature (175 °C), the packaged chip is essentially stress free. However, great residual stress will arise in the packaged devices when they cool down to ambient temperature (25 °C) due to the CTE mismatches among the components. The MEMS die suffers compressive stress, whereas the molding compound is subjected to tensile stresses since the CTE of the sensor die is much lower than the one of the molding compound. This diversification can result in high residual stress and warpage in the entire packaged device, leading to obvious variation of zero-offset and sensitivity. Krondorfer et al. [67] conducted a systematic study about the effects on pressure sensor from chip adhesion and molding. After the chip was attached onto the lead frame, the device behaved a TCO of 51.2% and TCS of −31.6% when the temperature changed from −7.5 °C to 125 °C. The values were 95.6% and −32.7% after molding. The compound molding brought a great effect to the sensor zero-offset, not only to the voltage value (from 0.41 mV to −43.6 mV) but also to the TCO (nearly two times larger). Though TCS was not greatly changed by molding in the same temperature range, but the absolute value still changed by about 20%. Also, the zero-offset at high temperature (125 °C) was much smaller than the one at low temperature (−7.5 °C), proving the stress decrease when the operation temperature was close to molding temperature.

For chip adhesion, the involved materials include substrate board, adhesive and sensor chip. The substrate board features large CTE difference with silicon-based chip and the adhesive layer, which connects the chip and substrate and acts as a buffer to accommodate the thermal residual stress. Optimization of thickness, Young’s modulus for adhesive usually plays an important role in chip mount. Generally speaking, thicker adhesive with low Young’s modulus generates lower residual stress and smaller zero-offset as well as smaller temperature drift to the sensor [68]. Overflow of adhesive is another common phenomenon when attaching the die onto the adhesive layer, which may impact the distribution of residual stress and further affect the output of sensors. Results show that the adhesive overflow can lead to 10% increase of thermal drift to the accelerometers [69]. Moreover, the viscoelastic behavior of PCB material (FR-4) also contribute to the thermal instability of piezoresistive sensors [70].

Analysis of compound molding is much more complex. The packaged device contains several elements and materials, making it difficult to establish an accurate analytical model. Thus, finite elements method (FEM) becomes the prior choice for researchers to simulate the packaged sensor and to improve the packaging proposals. By the process simulation, it has been shown that most of the thermal stress on the sensor chip is generated during the cooling process, and the stress during curing process can be neglected for its relative low modulus. According to the results reported by Kim et al. [42,71], the modulus, CTE and glass transition temperature *T_g_* are the key parameters for compound material selection. Materials with low modulus, low CTE and low *T_g_* can be the candidates to limit the thermal stress on sensor chip. Also, the material of compound should be treated as a viscoelastic not an elastic one, as the purely elastic model will lead to an exaggeration of the thermal stress in simulations.

### 3.5. Failure of p-n Junction and Ohmic Contact

When the operation temperature is above 150 °C, the p-n junction, formed by Born diffusion or implantation in n-type silicon, will display current leakage and form a reverse current through the junction [72]. The reverse current has a great influence on the thermal drift of output of Wheatstone bridge, leading to a thermal drift curve more complex than the parabola reported in [49]. However, the reverse current increases rapidly beyond 50 °C and its influence can be neglected when the temperature is beneath that value.

The high-temperature problem also occurs in the ohmic contact between metal electrode and doped silicon. The metal ohmic contact electrode made of Al, Cr/Au or Ti/Au cannot work at high temperature since the metals will diffuse into the silicon substrate and form a metal-doped silicon layer at the interface, significantly increasing the contact resistance [73].

Considering all the abovementioned inducements of thermal-performance instability, the residual stresses from adhesion and compound molding have the greatest impact on sensor performance, which are induced by the differences between material CTEs in these processes. Meanwhile, the influence of the membrane is considerable for the devices with thin/tiny sensing elements, such as the micro-pressure sensor with thin diaphragm. Some causes have been well studied, such as the temperature dependence of silicon piezoresistance, failure of p-n junction and ohmic contact between metal and silicon at high temperature. The thermal-performance instability of piezoresistive sensors is a consequence of the comprehensive influence of multiple factors, and the improvement measures should be proposed based on the particularities of each device.

## 4. Improvement in Thermal-Performance Stability

### 4.1. Keeping the Temperature Constant

**Figure 8:**
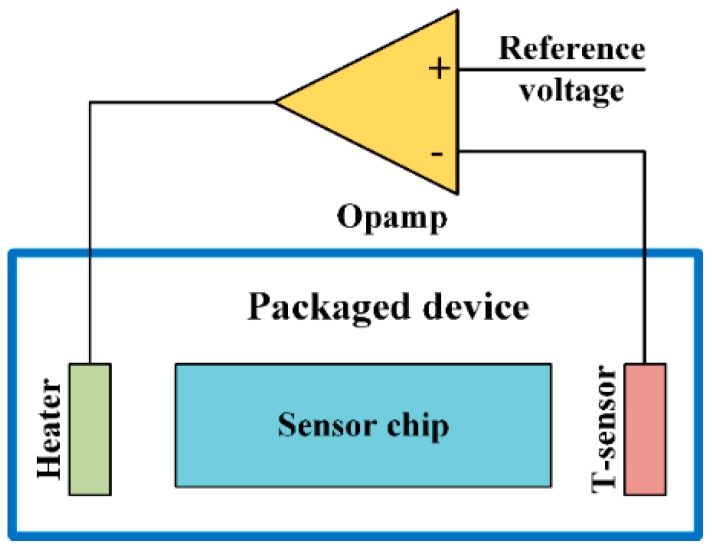
Schematic diagram of the temperature control system.

It is the temperature variation that causes most of the instabilities of sensor performance, so the influences can be eliminated if the sensor working temperature can be kept constant. This scheme needs a heater to raise the temperature, a temperature sensor (T-sensor) to monitor the real-time temperature and an amplifier to construct a closed temperature control loop. The schematic diagram of constant temperature control system can be found in Figure 8. A reference voltage is determined according to the output of T-sensor at the reference bias temperature. When the inner temperature of the device is lower than the bias one, the differential amplifier will excite the heater until the difference between the reference voltage and the output of T-sensor is balanced. If the output signal of T-sensor is equal to or higher than the reference one, the amplifier will stop the power supply. With this closed-loop system, the operation temperature of the sensor can be controlled at the bias temperature even though the ambient temperature changes. Thus, the sensor performance can be maintained over the temperature range from room temperature to the bias one. However, the bias temperature should be higher or equal to the maximum ambient temperature to ensure the system effectiveness.

Many efforts have been invested to implement this scheme. The pressure sensor in NASA’s Mars Pathfinder held a constant working temperature in the harsh environment of space, featuring a resolution better than 0.1 Pa with an accuracy of 3 Pa [74]. However, this device had a mass of approximately 500 g and consumed about 250 mW of continuous power, which brought a great burden to the space explorer [75]. Bruyker and Puers bonded the glass with heater and T-sensor electrodes onto the silicon diagram with piezoresistors to construct a thermostatic pressure sensor [76,77]. With the help of carefully designed circuit system, sensor TCO and TCS were reduced by more than 40 dB (two orders of magnitude) with respect to the non-controlled case over the temperature range of 0–40 °C with a power consuming of 150 mV. Lee et al. [78,79,80] integrated the T-sensor and microheater into a tri-axis accelerometer chip to keep the piezoresistor’s temperature at 300 °C. As a result, the dependence on temperature variation was much reduced over the range from room temperature to 300 °C: the temperature drift of sensitivity was decreased from 30% to 8%. Meanwhile, the measurement sensitivity was reduced about 28% compared with the value without temperature control. Moreover, the zero-offset was enlarged by about 14 times due to the high-level local thermal stress generated by non-uniform temperature distribution on the sensor beams. The power consumption of heaters was about 130 mV when rising the temperature from 27.1 °C to 300 °C.

The temperature control scheme provides a stable environment to piezoresistive sensors, whose performance dependence of temperature is much reduced. However, there are many deficiencies in the implementation of this proposal. First, the extra power consumption of the heater and T-sensor significantly limits the applications of this kind of sensor in Internet of Things (IoT), consumer electronics and other devices requiring low power consumption. Then, the temperature control system increases the complexity of sensor, leading to an increase in device cost and size, though the integration of heater and T-sensor has been realized. The reliability and lifespan may also be influenced by the long-term high temperature. Also, the local heating may cause high-level thermal stress in the sensor structure and enlarge the zero-offset.

### 4.2. Optimizing the Packaging Scheme

As mentioned in the section above, the residual stress induced by packaging processes is one of the main causes to sensor thermal instability. The chip adhesion and compound molding have become the focus of researchers and many optimized proposals have been conducted in sensor packaging [81]. Chen et al. conducted a series of simulation and experimental investigations on the optimization of wafer-level chip scale packaging (WL-CSP) for piezoresistive pressure sensors. Two different approaches, sacrifice-replacement and dam-ring, were modeled, simulated and tested [82]. In the sacrifice-replacement approach, WL-CSP with small polyimide thickness and large opening window produced small packaging induced stress [83,84]; in the dam-ring approach, large sensing channel opening was also preferred [85]. Lee et al. [86] composed a flip chip and flex circuit packaging technique, which used a spacer to support the silicon sensor chip and a solder bump to provide electrical connection with the flex circuit. This method reduced the contact area between sensor chip and substrate and thus degraded the packaging-induced thermal effect on pressure sensor. The FEM results showed that the novel design decreased the temperature-induced output variation of 10 psi from 0.222 mV to 0.035 mV and also maintained the sensor sensitivity. However, the pressure on sensing diagram tend to separate the chip from the substrate and the reliability problem might arise if this design is used in high pressure measurement. Schröder et al. [87,88] presented a wire bonding based packaging approach for inertial sensors. The sensor chip was exclusively attached to the package frame by bonded wires on both front and back sides. The symmetric mount facilitated significant reduction of thermal stress. However, the supporting stiffness greatly depended on the number of bonded wires, and partial failure of wires will lead to complete failure of the packaged device. Moreover, the effect on sensor dynamic performance should also be considered.

For the optimization of compound molding, many researchers have dedicated their efforts to its model construction, simulation and experimental verification. The viscoelastic effect and stress relaxation behavior are obtained by utilizing dynamic mechanical analysis [89] and sensor-aided measurement [43,90]. With the carefully measured material parameters, FEM models are constructed and verified. Accurate assessment of packaging stress effects on sensors are adopted and the zero-offset can be reduced by more than 80% [66]. Meantime, effects form different parameters are studied and optimization selection of packaging materials is fulfilled [71]. The residual stress in wafer anodic bonding can be limited by ameliorating the pressure electrodes [62,63] and non-isothermal bonding [91]. Using the four-dot adhesion of sensor chip [92] and filtering the silicon oil and decreasing its amount in the packaging of an engine oil pressure sensor [93] will also help a lot in the sensor performance improvement.

The effects of packaging play the dominant position in sensor thermal instability, and many works have been done in the packaging optimization. However, the stress from packaging still cannot be significantly reduced, and the improvement in packaging usually requires a large number of specific processes, which is not very compatible with the practical production.

### 4.3. Temperature Compensation

#### 4.3.1. Compensation by Resistors

**Figure 9:**
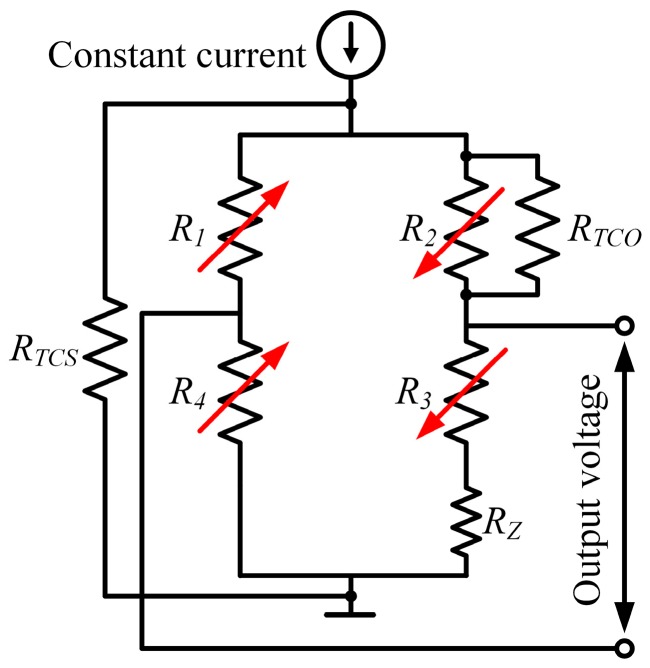
Schematic diagram of the compensation by resistors.

The effects from temperature variation, regardless of source, eventually perform as the output variation of Wheastone bridge. Therefore, incorporating extra proper resistors into bridge circuit to cancel out the influence is a plan that easy to think of [8]. A typical compensation network is illustrated in Figure 9. *R*_z_, *R*_TCO_ and *R*_TCS_ are the resistors for compensating zero-offset, TCO and TCS, respectively.

In order to facilitate the zero-offset compensation, the bridge circuit can be designed into a half open loop (also called as five-interface structure). Setting *V*_0_ as the zero-offset voltage and *I_i_* the excitation current, the resistance of *R*_z_ can be approximately calculated as [94]:

(13) $$R_{Z}\approx \frac{4\left|V_{0}\right|}{I_{i}}$$

With the approximate value and mite adjustment, the zero-offset can be well controlled. If *V*_0_ > 0, *R*_z_ should be located in the bridge arm of *R*_3_ as shown in Figure 9; otherwise, *R*_z_ should be in the arm of *R*_4_. However, the compensations of TCO and TCS will incorporating other resistors and *R*_z_ is usually calculated and connected after the TCO and TCS compensations are finished. The compensation of TCO is fulfilled by paralleling a resistor *R*_TCO_ at one bridge arm and its resistance can be expressed as [94]:

(14) $$R_{TCO}\approx \frac{I_{i}\left(R_{T2}^{2}-R_{T1}^{2}\right)}{4|\Delta V_{0}|}$$

where Δ*V*_0_ is the difference between the zero-offset at temperatures of *T*_1_ and *T*_2_, *R_T_*_1_ and *R_T_*_2_ are the average resistance of all the resistors at *T*_1_ and *T*_2_, respectively. When Δ*V*_0_ > 0, *R*_TCO_ should be paralleled with *R*_4_; otherwise, *R*_TCO_ should be paralleled with *R*_1_. The compensation resistor for TCS can be determined as [95]:

(15) $$R_{TCS}=\left|\frac{TCS_{0}}{TCS_{0}+TCR_{B}}\right|R_{B}$$

where *TCS*_0_ is the TCS of uncompensated bridge and *R*_B_, *TCR*_B_ are the bridge resistance and its TCR. *TCS*_0_, *R*_B_ and *TCR*_B_ can be easily obtained from experiments. All the compensation resistors should feature very low temperature coefficient of resistance, making the compensation effective and reliable.

#### 4.3.2. Compensation by Programs

With the development of microprocessor techniques, programing compensation for piezoresistive sensors is getting more and more attention, especially for high-accuracy devices. With the on-board memory, correction for temperature drift can be realized by software and achieves sufficient improvement in accuracy compared to hardware trim techniques. The look-up table method and polynomial fitting method are the most commonly used forms in the initial stage of digital compensation. The look-up table method needs adequate calibration data to achieve high accuracy, which is directly correlated to the experimental work and memory capacity [96]. The polynomial fitting method fits the calibration data with formulas with different orders. Šaponjić constructed a second-order polynomial with the help of microprocessor to fulfill temperature compensation and output linearization of pressure sensor, which improved the accuracy over a small measuring range of 0–200 kPa at temperatures from 0 °C to 70 °C [97]. IEEE 1541.2 recommended the Taylor expansion as the general approach to describe sensor characteristics. The piecewise-fitted method in different temperature regions get a better similarity coefficient and improved the TCO by an order of magnitude [98]. Meantime, high-order temperature compensation model is another method of improving sensor temperature independence and linearity [99]. However, the abovementioned methods suffer the following disadvantages: first, the fitting accuracy is severely limited by the number of experimental point and coverage of measurement range and application temperature. Improper selection of experimental point and coverage will lead to a result that the fitting formula cannot accurately describe the sensor characteristics, which could decrease the compensation efficiency. However, the sufficient data come from a large number of calibration experiments, which are very time-consuming and laborious. Second, compensation efficiency also depends on the fitting model. Low-order polynomial cannot completely characterize the experimental curve, but polynomials with higher order will consume more processor resources, causing reduction in real-time performance. Last, the models in above compensations are lack of universality. The models for each device should be constructed based on its experimental results, which limits the applications of these schemes in practice.

**Figure 10:**
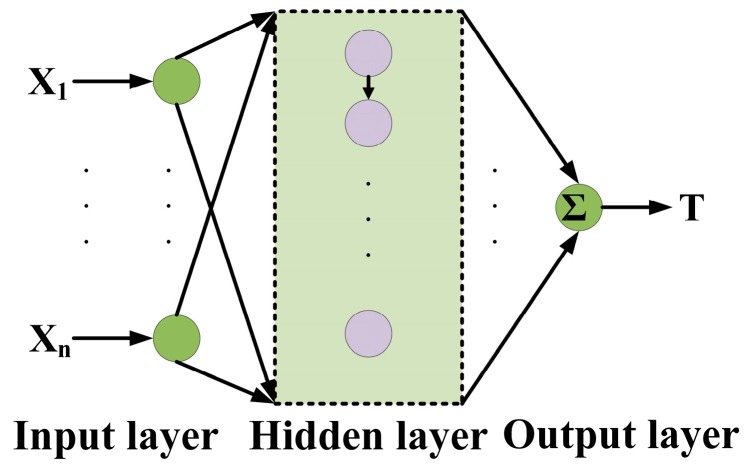
Basic structure of ANN.

Therefore, compensation programs based on intelligent algorithm become a favorite of practical applications. Artificial neural network (ANN)-based signal conditioning is an alternative approach to compensate the temperature drift of piezoresistive sensors over the chosen range of ambient temperature values. Due to the adaptability and generalization capability, ANN can be trained with enough information to learn any available function, coupled with judiciously selected neural models. This self-learning ability eliminates the usage of complex and difficult mathematical analysis, which is dominant in the polynomial fitting methods. The basic structure of ANN can be found in Figure 10. Usually an ANN has three layers including input, hidden and output layer. The input layer is used to receive the original experimental results; the hidden layer contains several neurons to pursue minimum error, and the training of hidden layer can be implemented based on different algorithms, such as least mean square (LMS) [100], support vector machine (SVM), variable coefficient regression (VCR), extreme learning machine (ELM) [101] etc. Meantime, ANN can be implanted in either digital or analog circuits, and ASIC techniques has been incorporated into the intelligent compensation [102,103].

Compensation by hybridizing resistor and program approaches is also reported. Hardware is usually used to adjust the zero-offset and output span of sensor, and program is used to enhance the thermal stability, linearity and accuracy [104]. The hybridism utilizes the advantages of different methods, making the compensation more effective. Though possessing excellent results, compensation by program still encounters many difficulties in practical applications. The large number of calibration data require a lot of time and manpower. Meantime, extra elements, such as amplifiers, resistors and microprocessors, will occupy more space, consume more power and cost extra money.

#### 4.3.3. Compensation by Dummy Units

In piezoresistive sensors, normal piezoresistors is used to sensing the target-induced stress. Different from the normal piezoresistors, the dummy unit, also composed by piezoresistors, just senses the influence form temperature variation. Introducing dummy unit into the sensor can be an effective scheme for temperature compensation if the thermal disturbs can be eliminated by the special bridging arrangement of both kinds of piezoresistors. Dummy unit compensation have been realized for cantilever-based piezoresistive sensors. The dummy cantilever (also called reference cantilever), co-fabricated in close proximity to the actual sensor, is expected to feature a similar temperature-dependent behavior to the functional one. The piezoresistors on each cantilever can form a Wheatstone bridge to reduce the temperature sensitivity [105]. Moreover, the sensor sensitivity can be further improved if a full Wheatstone bridge is integrated in each cantilever [106]. However, there are still some problems in the compensation by two co-fabrication cantilevers. First, the couple of cantilevers, though very close to each other, are still physically separated from each other, inducing a second-order spatial variations in the external thermal environment. Thus, a single cantilever compensation scheme was proposed [107]. In the cantilever, sensing piezoresistor was arranged along the <110> orientation of silicon and obtained the largest piezoresistive coefficient, but the dummy piezoresistor was along <100> orientation with lowest piezoresistive coefficient. With these specially arranged piezoresistors, the novel compensation scheme decreased the TCO by about 94% compared to the co-fabrication scheme. Nevertheless, the measurement sensitivity was only 25% of the full bridge propose, for only one sensing resistor existed there. On the other hand, the difference between the thermal inertia of two cantilevers also causes different zero-offset when the two cantilevers were excited by voltage, which could be relieved by the stripe patterned immobilized layer [108].

Dummy unit compensation can also be used in the compensation of pressure sensors. One way to introduce a dummy unit is doping the piezoresistors in the substrate and the resistance only changes with temperature [109,110,111]. And this method is equally effective for accelerometers [112]. Another method is possessing a ventilation micro-hole and buried micro-channel to introduce detected pressure into the pressure reference chamber, making the dummy unit insensitive to applied pressure. This dual-unit scheme can be utilized to eliminate the effect from the residual stress in passivation layers on silicon diagram [39].

### 4.4. Mechanical Isolation of Packaging Stress

Stress from chip adhesion and compound molding has been proved to be the main cause for the thermal-performance instability of piezoresistive sensors. Thus, the proposal that isolating piezoresistors from packaging stress by exquisite design of sensor structure is put forward [113], and many devices with mechanical stress isolation structure have been developed. Generally speaking, this kind of sensor transforms the packaging stress into the deformation of its isolation structure, which prevents the interference from packaging stress to piezoresistors.

The mechanical stress isolation can be conducted in two ways: isolating the whole sensing structure and just isolating the piezoresistor region. The former scheme usually introduces a yielding mechanism around the whole sensing structure and separates it from the adhesion regions of sensor chip. In the pressure sensor developed by Spiering et al., the circular corrugated decoupling zone, with a high tangential stiffness but low radial stiffness, transformed the extra stress loads into local deformations with a small impact on the inner edge of the zone, reducing the influence from packaging by several orders of magnitude [114,115]. In the piezoresistive pressure sensor fabricated by microholes interetch and sealing process [39,116], the compact cantilever-shaped packaging-stress-suppressed suspension (PS^3^) was on-chip integrated surrounding the pressure-sensing structure. The TCO was about 15 times better than that of the sensor without PS^3^ and nearly no loss in measurement sensitivity was observed. A similar cantilever-shaped yielding structure can also be found in the package-friendly tri-axis accelerometer developed by Hsieh et al. [117]. The stress isolation guard-ring (GR) was anchored to the substrate through the connection cantilever, suspending the whole sensing structure [118]. The yielding structure can also be realized in the twin-mass accelerometer when the perpendicular outer beams are substituted by lateral external beams [119]. The partially isolation scheme, mainly preventing piezoresistors from packaging stress, can be traced back to the year of 1990. Hälg et al. separated the section containing piezoresistors from the substrate by a free space, forming a certain degree of mechanical isolation of packaging stress [120]. The surrounding mass structure is also an effective scheme for reduction in thermal stress [121]. In that structure, a central support portion is attached onto the substrate and the suspended surrounding mass is connected to the central portion by four beams, acting as the proof mass. Thereby, the piezoresistors on the connecting beams are isolated from the adhesion area [122]. Thermal stress relaxation with ring-shaped beams in the accelerometer also play an isolation role [123]. One or more rings are arranged between the piezoresistors and outer adhesion area, so that the thermal stress will be relaxed in the ring region before it arriving at piezoresistors.

**Figure 11:**
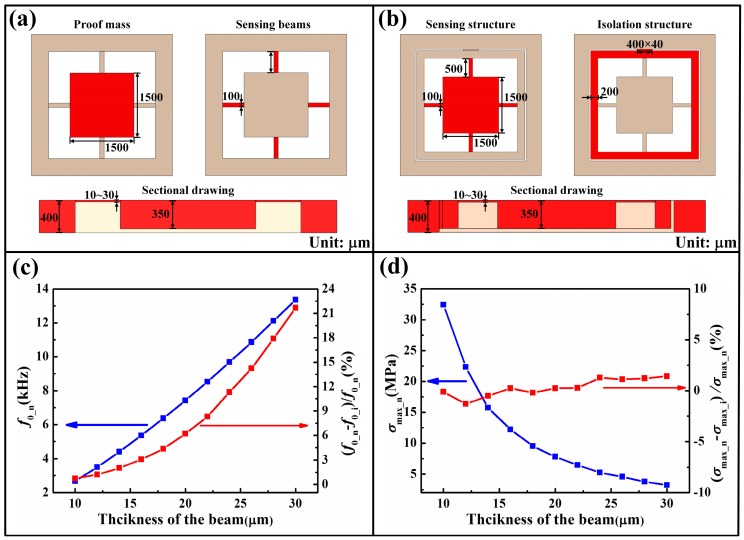
Models and simulation results: (**a**) Model and dimensions of the sensor without GR; (**b**) Model and dimensions of the sensor with GR; (**c**) Simulation results of resonant frequency; (**d**) Simulation results of maximum normal stress in the beams.

The mechanical isolation approaches, no matter whether entirely or partially isolating, may not be very applicable in the high-frequency devices. Most of the abovementioned sensors with mechanical isolation structure feature a low resonant frequency, and their static and dynamic performances are not greatly influenced. However, that situation will change if the isolation structures are incorporated into high-frequency sensors. Herein, cross-beam accelerometers with/without GR structure are modeled and simulated to study the effects from GR. The two models and their typical dimensions are depicted in Figure 11a,b. The material parameters of silicon are set as: Young’s modulus *E* = 166 GPa, density Δ = 2331 kg/m^3^ and Poisson’s ratio ν = 0.27. The applied acceleration is 50 g. *f*_0_n_ is the resonant frequency of structure without GR, and *f*_0_i_ is the resonant frequency of structure with GR. *σ*_max_n_ is the maximum normal stress induced by the applied acceleration in the cross beams without GR (characterizing the measurement sensitivity), and *σ*_max_i_ is for the beams with GR. It can be seen in the curves that the resonant frequency increases with the increment of beam thickness, but the maximum stress significantly decreases, corroborating the interdependent relationship between frequency and sensitivity. Meantime, *f*_0_i_ is more and more lower than *f*_0_n_, indicating that the introduction of GR has a great influence on the dynamic characteristics. However, the difference between *σ*_max_n_ and *σ*_max_i_ remains at a relative small value, confirming the low impact of GR on measurement sensitivity. The utilization of GR (or other mechanical isolation structures) in high-frequency sensors needs detailed evaluation and careful design to ensure the excellent comprehensive performance of sensors.

In addition to the abovementioned measures, some other methods have also been proposed to enhance the immunity to high temperature. First, proper doping concentration is chosen in the formation of piezoresistors to cut down the temperature dependence of silicon piezoresistance. That dependence obtains a negative relationship with concentration when the value lies in the range of 3 × 10^17^~3 × 10^19^ cm^−3^; when the concentration is higher than 3 × 10^19^ cm^−3^, the piezoresistive coefficient appears nearly no change with temperature [47]. Therefore, many designs choose higher doping concentration for piezoresistors to get stable piezoresistance, though the measurement sensitivity is also reduced for the small piezoresistive coefficient at high concentration. Then, SOI wafer is utilized to separate the p-n junction from silicon substrate. The reverse current is eliminated and the operation temperature limitation of sensors can be promoted from about 150 °C to above 250 °C [28]. Also, some wide gapped materials, e.g., SiC, are also used in piezoresistive sensors to heighten the working temperature. Last, thermal stable electrodes with multi-metal layers are applied to prevent the failure of ohmic contact. The new electrode made of TiSi_2_/Ti/TiN/Pt/Au still maintains its good contact characteristic at 500 °C, adopting a more excellent performance to the SOI piezoresistive sensors [73].

The main schemes for enhancing thermal-performance stability are summarized in Table 1. Considering the efficacy, the constant temperature method, microprocessor-aided compensation, dummy compensation and mechanical isolation feature excellent performance in the amelioration of sensor instability. The first three methods can handle nearly all the causes, so they behave to good effect. Meantime, the mechanical isolation method mainly treats the packaging stress and also has an excellent efficacy. This phenomenon proves that packaging stress is the most important cause of sensor performance instability, which also can be confirmed by the limited roles of the methods treating other single factors. When considering the whole sensor system and its applications, many factors, e.g., system complexity, power consumption and system integration, should also be investigated when selecting the improvement methods. The constant temperature method and programing compensation need additional elements to construct the whole system, increasing the system complexity, cost and power consumption. Dummy unit compensation and mechanical isolation introduce exquisitely designed structures into the sensor to fulfill the enhancement target, bringing potential changes to sensor performances. The residual stress from chip packaging is the main factor for thermal instability, but the optimization still cannot completely eliminate the stress and effective and adaptable packaging approaches are needed. Comprehensively, the mechanical isolation may be the excellent candidate if the high-frequency problem is solved by more exquisite designs. Definitely, combination of different schemes is highly recommended, but the application requirements and limitations should be fully considered. Moreover, utilization of new materials and fabrication processes can also be a good solution to thermal instability.

## 5. Conclusions

This paper presented a comprehensive review of the matters associated with thermal-performance instability in piezoresistive sensors. The instability is the combined effect of many factors. To understand the physical mechanism behind the temperature dependence of sensor characteristics, the pertinent inducements are categorized as different aspects, including temperature-dependence of silicon piezoresistance, membrane/fabrication/packaging-induced residual stress and possible failures at high temperature. Among them, the influences from chip packaging account for the main part, about 80%–90%, of sensor performance variations. Then, available improvement measures from literatures are concluded. Their basic principles, realization methods, applicable objects and merits/demerits are summarized. Though the utilization of temperature control system, compensation algorithm and dummy unit can degrade the targets by about 90%, but additional components and resources are also needed. Meantime, the packaging-friendly sensor chip with mechanical isolation structure can be the excellent solution, but careful design is required when proposing a high frequency sensor. The development in thermal-performance stability of piezoresistive sensors is still being excited by new materials, fabrication techniques and other novel enhancing approaches.

## Figures and Tables

**Table 1:** Summary of available measures for improvement of thermal-performance instability in piezoresistive sensors.

Improvement Scheme	Implementation Method	Referred Paper	Target Inducement	Efficacy ^1^	Comment
**Constant temperature**	Maintaining the device temperature at a preset value by the closed-loop system including T-sensor, heater and differential amplifier	[76,77,78,79,80]	Nearly all causes	>90% in TCS and TCO [76]	**Adv ^2^:** Immune to almost all effects from temperature variation **Disadv ^3^:** Local thermal stress, extra elements, large power consumption, problems in reliability and life time.
**Packaging optimization**	Non-isothermal bondingmultiple pointed pin electrodelow temperature bonding	[62,63,91]	Residual stress in anodic bonding	53% in wafer bow [63]	**Adv:** null **Disadv:** Process incompatibility with existing equipment
	Optimizing the dimensions of polyimide thickness and open window	[82,83,84,85]	Residual stress from packaging	65.7% in TCO [82]	**Adv:** Simple and effective, favorable compatibility **Disadv:** Narrow application range
	Parametric analysis and simulation about the effects from materials	[42,68,71]	Residual stress from packaging	48.3% in TCS [68]	**Adv:** Providing a guideline for material selection **Disadv:** The optimized parameters could not be achieved in practice.
	Mounting the sensor chip by symmetrically-bonded wires	[87,88]	Residual stress from packaging	73.6% in chip warpage	**Adv:** Nearly no residual stress **Disadv:** Low supporting stiffness, a great number of invalid wire bonding, process incompatibility
**Compensation**	Series or parallel connection of resistors	[8,94,95]	Temperature dependence of piezoresistance	Null	**Adv:** Simple **Disdv:** Low accuracy, limited effect, inflexible
	Microprocessor-aided compensation with various algorithms	[96,97,98,99,100,101,102,103]	Nearly all causes	94.9% in accuracy [101]	**Adv:** High accuracy, high universality, intelligent **Disadv:** Calibration experiment taking time and effort, increasing complexity and cost
	Eliminating thermal stress by dummy units	[39,105,106,107,108,109,110,111,112]	Nearly all causes	84.2% in TCO [39]	**Adv:** Simple **Disadv:** Influencing sensor measurement performance, enlarging the size of sensor chip
**Mechanical isolation**	Transforming the packaging stress into deformation of yielding structures	[113,114,115,116,117,118,119,120,121,122,123]	Residual stress form packaging	93.8% in TCO [116]	**Adv:** Packaging-friendly, without extra elements or system **Disadv:** Influencing dynamic performance of sensors
**SOI wafer/multi-layer metal**	Enhancing the immunity to high temperature	[28,47,73]	Failure of p-n junction and ohmic contract	Null	**Adv:** null **Disadv:** Process incompatibility

^1^ Efficacy = the descent percentage of the target parameters; ^2^ Adv = Advantages; ^3^ Disadv = Disadvantages.
